# Pulsed focused ultrasound enhances mesenchymal stem cell homing to skeletal muscle in a murine model of muscular dystrophy and homing was suppressed by Ibuprofen

**DOI:** 10.1186/2050-5736-3-S1-P69

**Published:** 2015-06-30

**Authors:** Ben Nguyen, Scott Burks, Saejeong Kim, Michele Bresler, Pamela Tebebi, Joseph Frank

**Affiliations:** 1 National Institutes of Health, Bethesda, Maryland, United States

## Background/introduction

Homing of iv-infused stem cells to diseased tissues may be critical for cell therapies and is frequently an obstacle to successful cell therapy. We have previously shown that molecular responses from the mechanotransduction effects of pulsed focused ultrasound (pFUS) in normal murine skeletal muscle generate a “molecular zip-code” consisting of local increases in chemoattractants (cytokines, chemokines, cell adhesion molecules) that induced MSC homing, potentially improving cellular therapies for regenerative medicine. This study investigated whether pFUS could also enhance MSC homing to dystrophic skeletal muscle in a muscular dystrophy (MD) mouse model. Stem cell therapies for MD are promising, but have been hampered by poor cell homing and the need for direct injections that are invasive and ultimately, impractical clinically. Since molecular signals drive cell homing following iv injection, drugs used to treat MD could potentially interfere with pFUS-enhanced homing and undermine cell therapies for MD.

## Methods

**Figure 1:**
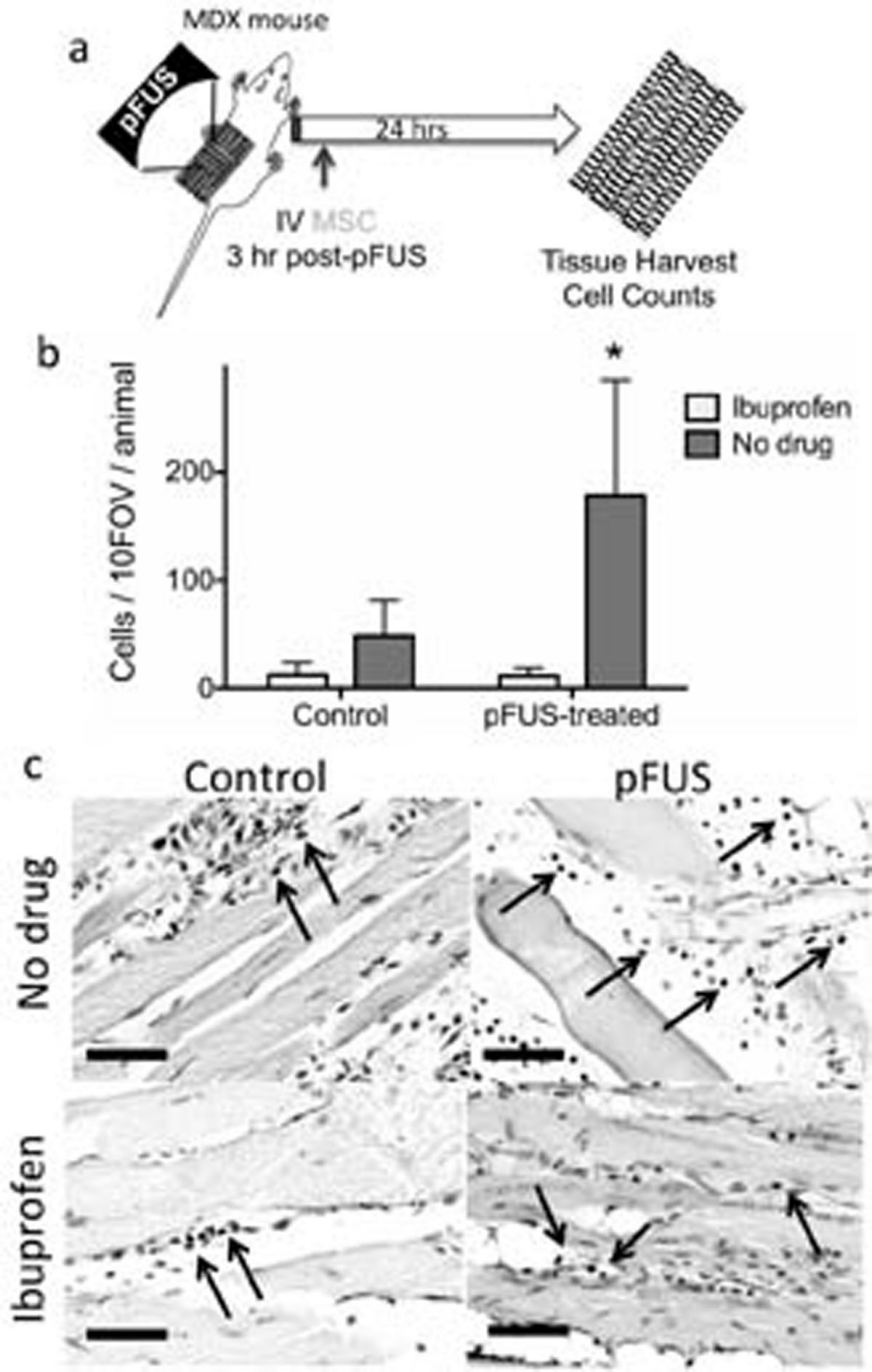
Pulsed focused ultrasound enhances homing of mesenchymal stem cells to dystrophic muscle. a) experimental design. b) quantification of MSC homing. c) representative immunohistochemistry (MSCs indicated by arrows).

MDX mice (9 weeks) received unilateral pFUS (1MHz, 5MPa, 10 ms pulses, 5% duty cycle, VIFU 2000) to hamstrings. Some mice were pretreated with ibuprofen (nonspecific cyclooxygenase [COX] inhibitor; 30mg/kg, po) or saline 15min pre-pFUS. Mice were iv injected with 106 human MSC 3hr post-pFUS. Hamstrings were harvested 24hr post-injection, MSC were detected by immunofluorescence and compared to untreated contralateral hamstrings (Fig 1a).

## Results and conclusions

Minimal MSC homing was observed to skeletal muscle in MDX mice, but pFUS treatment enhanced MSC homing by ~15 fold (p<0.001) (Fig 1b). Numerous MSCs were observed in interstitial spaces between myofibers (Fig 1c). Furthermore, pFUS failed to increase MSC homing when mice were pretreated with ibuprofen (i.e., MSC homing to pFUS-treated hamstrings was not significantly different (p>0.05) than control contralateral hamstrings). This suggests that COX2 signaling is critical for pFUS-enhanced MSC homing to dystrophic muscle. pFUS-enhanced MSC homing to dystrophic skeletal muscle may represent an ideal platform to deliver MSC and other therapeutic cells (e.g. myogenic stem cells) to tissues that are perpetually in a sub-acute or chronic inflammatory states not conducive to homing of therapeutic cells. Furthermore, anti-inflammatory drugs, including ibuprofen, are currently a standard-of-care for treating the progression of MD. Ibuprofen suppression of pFUS-enhanced MSC homing in MD reveals potentially negative drug/host interactions that are currently uncontrolled for in clinical cell trials and will need to be accounted for when developing future MD cell therapies.

